# Human *SLC4A11* does not complement *BOR1* or support borate transport in *Saccharomyces cerevisiae*

**DOI:** 10.17912/micropub.biology.000605

**Published:** 2022-07-20

**Authors:** Jean L. Beltran, Richara K. Bain, Marie J. Stiefel, Alexis S. McDonnell, Natalie N. Carr, Bryan H. Thurtle-Schmidt

**Affiliations:** 1 Department of Biology, Davidson College, Davidson, NC 28035, USA

## Abstract

Borate is an essential micronutrient in plants regulated by borate transporters, which also protect both yeast and plants from toxically high levels of borate and share homology with the human SLC4 transporters. SLC4A11 is linked to congenital hereditary endothelial dystrophy and was initially reported to transport borate before subsequent studies rebutted this conclusion. To better understand the transport activities of purported borate transporters, we tested the ability of *SLC4A11* and eleven borate transporters from *A. thaliana* and *O. sativa* to complement a *BOR1* deletion in *S. cerevisiae* . We show that *AtBOR4* , *AtBOR5* , *AtBOR7* , *OsBOR2* , and *OsBOR3* can each complement *ScBOR1* , while the rest of the transporters tested do not rescue growth. Additionally, quantification of intracellular borate content demonstrates that SLC4A11 does not export borate in yeast, supporting studies that its transported substrate is not borate.

**Figure 1. Testing borate transport activity by genetic complementation and borate quantification assays:**
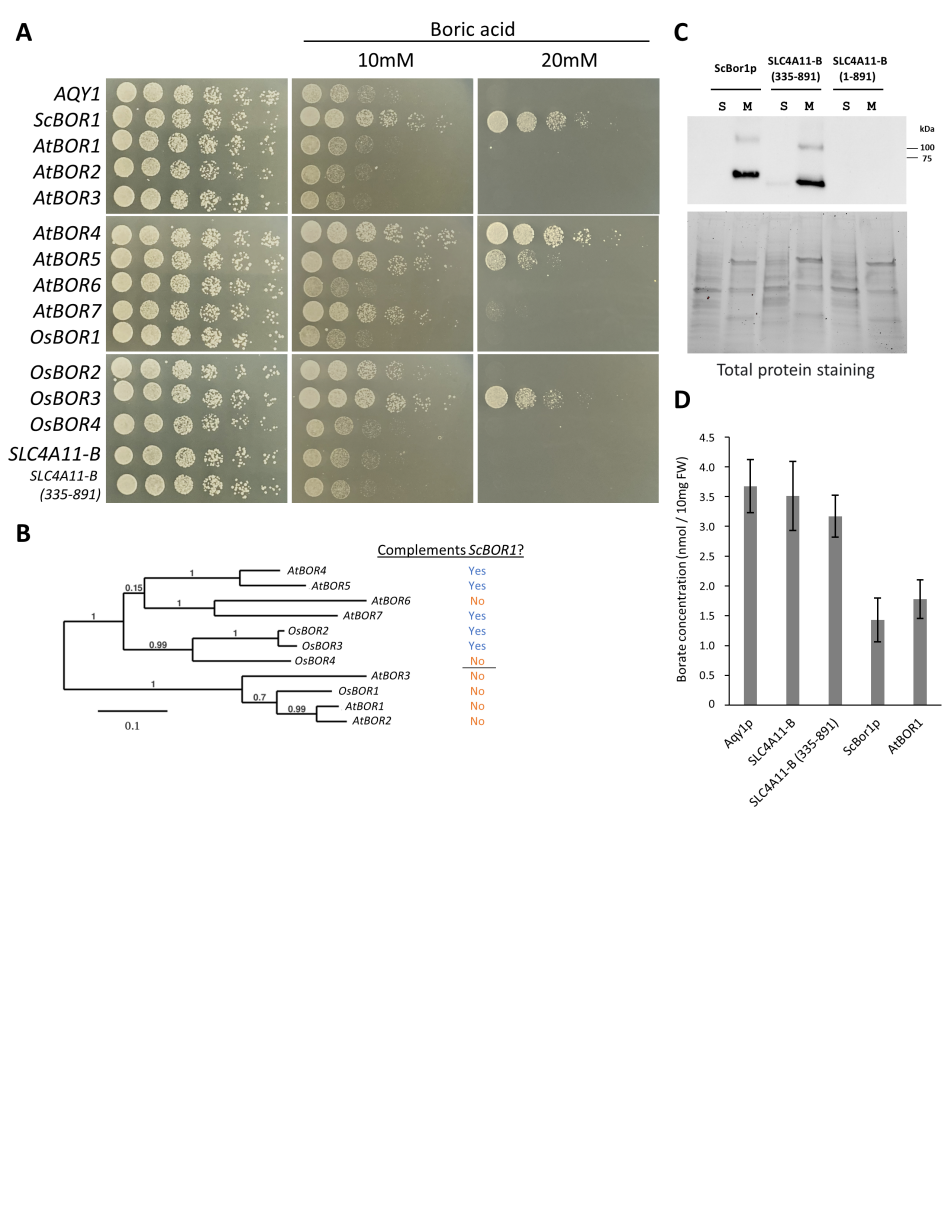
( **A** ) Plasmids encoding the specified transporter genes were transformed into *bor1* deletion cells and the ability of each to rescue growth was tested by plating fivefold serial dilutions onto plates containing CSM-His selective media, 2% raffinose, 0.05% galactose, and the indicated boric acid concentrations. Plates were incubated at 30°C and imaged after 4 days. ( **B** ) Phylogenetic relationships of *A. thaliana* and *O. sativa* borate transporter genes. Indicated to the right of each gene name is a “Yes” or “No” to indicate whether the gene complements *ScBOR1* . ( **C** ) Western immunoblotting analysis of His-tagged SLC4A11-B and ScBor1p. ( *Top* ) “S” corresponds with supernatants and “M” with pelleted membranes collected from a high-speed spin of cell lysates. Samples were analyzed with an anti-His _6_ -tag antibody. ( *Bottom* ) Total protein stain-free gel imaging of 8.75μg protein per lane served as loading controls. ( **D** ) Quantification of borate efflux activity in yeast cells expressing each indicated protein. Intracellular borate contents are reported as nmol per 10mg fresh weight (FW) of yeast cells after 90 min incubation of cells in 1mM boric acid. Error bars represent 95% confidence intervals for n = 8 biologically independent experiments.

## Description

In *S. cerevisiae* , the borate transport protein Bor1p mediates resistance to toxically high levels of borate (Takano et al., 2007). In plants, the role of borate transport is more complicated given the organism’s need to prevent borate toxicity while also maintaining high enough levels of borate as a micronutrient that supports growth (Warrington, 1923). As a result of these complex needs, the borate transporter family has expanded in plants, with seven borate transporters in *Arabidopsis thaliana* ( *AtBOR1* through *AtBOR7* ) and four borate transporters in *Oryza sativa* ( *OsBOR1* through *OsBOR4* ). The human transporter SLC4A11 was initially characterized as a borate transporter (Park et al., 2004), but subsequent studies have rebutted this finding (Ogando et al., 2013; Kao et al., 2015; Loganathan et al., 2016b). Understanding SLC4A11 function and substrate transport activities is important because mutations in SLC4A11 have been shown to cause corneal diseases like congenital hereditary endothelial dystrophy (Vithana et al., 2006).

We examined borate transport using genetic complementation assays in a *S. cerevisiae* strain where we have knocked out *BOR1* . We tested complementation with all seven borate transporters in *A.* *thaliana* ( *AtBOR1* through *AtBOR7* ) and all four in *O.* *sativa* ( *OsBOR1* through *OsBOR4* ). We also tested the full-length B isoform (also called v2) of human SLC4A11, which has been reported to be the most common of three isoforms found in the corneal endothelium (Malhotra et al., 2019). To test whether the SLC4A11 membrane domain alone can express and function in yeast, we also created an N-terminally truncated construct that removed the cytosolic domain and left intact its predicted membrane domain (residues 335-891, using the B isoform numbering). We challenged the yeast with either 0, 10, or 20mM boric acid and imaged plates 4 days after plating. Shown in Figure 1A are representative plates from at least three distinct biological replicates of this experiment for all 15 samples. For a negative control we chose a plasmid bearing *AQY1* , a yeast aquaporin gene that does not support borate transport (Nozawa et al., 2006). *AtBOR4* , *AtBOR5* , *AtBOR7* , *OsBOR2* , and *OsBOR3* can each complement yeast *BOR1* , with the *AtBOR7* and *OsBOR2* phenotypes most evident in the 10mM but not the 20mM boric acid condition. Interestingly, all five plant transporters that complement are found in one of two clades of borate transporters (Fig. 1B). Further studies will be necessary to determine whether this distinction illuminates important functional differences between the two clades.

Neither the full-length nor truncated form of human SLC4A11-B complements *ScBOR1* (Fig. 1A). The inability of both SLC4A11 constructs to rescue growth in our experimental conditions does not on its own establish an inability to transport borate, as a lack of expression in yeast could explain these data. To rule out this possibility, we performed Western immunoblot analysis with the full-length and truncated SLC4A11-B constructs, along with ScBor1p as a positive control. Total protein staining demonstrates the same amount of protein was loaded per sample, while a primary antibody recognizing His-tag residues reveals that full-length SLC4A11-B does not express, but that the truncated membrane domain construct expresses comparably well compared with ScBor1p (Fig. 1C). Surprisingly, our results are the inverse of a previous study in HEK293 cells that shows the full-length protein to express at the cell surface while the membrane domain in isolation expresses and functions poorly (Loganathan et al., 2016a). The weaker, higher molecular weight band is likely to be dimer, which has been previously observed to persist for borate transporters on SDS-PAGE gels (Flores et al., 2019). Both ScBor1p and the truncated SLC4A11-B are found localized to pelleted membranes but not to the supernatant corresponding to cytosolic proteins. Our results show that the inability of the truncated SLC4A11-B construct to complement *ScBOR1* cannot be attributed to a lack of expression.

It is possible that a transporter could export borate but at levels insufficient to complement *ScBOR1* and support yeast growth on plates containing boric acid. To test this possibility, we quantified the amount of intracellular borate in yeast cells expressing *ScBOR1* , *AtBOR1* , and our two *SLC4A11* constructs using a curcumin based colorimetric assay (Mohan and Jones, 2018). Cells were grown up in liquid culture, treated with 1mM boric acid for 90 minutes, and then their lysates were harvested and analyzed for borate content. Eight independent biological replicates were performed for each sample, along with standards used to generate standard curves to calculate borate quantities for each experiment. Data are presented normalized to cell pellet weights and with error bars representing 95% confidence intervals. As expected, our data show that cells expressing ScBor1p have lower borate amounts relative to the Aqy1p negative control. Interestingly, despite its inability to complement *ScBOR1* , *AtBOR1* shows borate export comparable to *ScBOR1* (Fig. 1D). Our data highlight a curiosity in the literature regarding the ability of *AtBOR1* to support transport borate in yeast: multiple studies have reported that *AtBOR1* cannot complement a *BOR1* deletion in yeast (Thurtle-Schmidt and Stroud, 2016; Miwa et al., 2007), and multiple other studies have reported that it can (Saouros et al., 2021; Yoshinari et al., 2021). Here we give detailed experimental conditions in which *AtBOR1* does not complement a *BOR1* deletion, but also show using the same media reagents that *AtBOR1* demonstrates robust borate transport activity by an alternative assay. Genetic complementation and borate quantification assays have both been used to study borate transporter phenotype and function in numerous studies, and our data here suggest that caution is warranted in relying too heavily on any one experiment to draw conclusions about borate transporter function.

The borate quantification data show both SLC4A11-B constructs display borate levels comparable to that of the Aqy1p negative control (Fig. 1D). These results suggest that expression of SLC4A11 in yeast does not result in borate transport. Previous studies indicating a lack of borate transport by SLC4A11 all relied on experimental methods that did not directly measure borate quantities, but instead relied on measuring outcomes predicted by borate transport, including cell acidification upon borate addition (Ogando et al., 2013; Kao et al., 2015) and cell swelling upon borate addition (Loganathan et al., 2016b). These studies report SLC4A11 to be an electrogenic H ^+^ (OH ^-^ ) transporter, and their data and ours here provide strong evidence that SLC4A11 does not transport borate. In sum, our data identify multiple *A. thaliana* and *O. sativa* borate transporters that can be further characterized in yeast and demonstrate that borate transport experiments in *S. cerevisiae* cannot be used to study the function of SLC4A11.

## Methods

Genetic complementation assay

A yeast strain (BTSY2) with a knocked out *BOR1* ( *MATα his3::GAL1-GAL4 pep4 prb1-1122 bor1::KanMX* ) was transformed with plasmids that differ only by the transporter they encode. In all experiments reported here, the plasmid backbone is pRS423 and the genes are under inducible control of the *GAL1* promoter and possess a C-terminal thrombin site and His _10_ tag. Overnight cultures were grown in media consisting of yeast nitrogen base (YNB+nitrogen), complete supplement mixture lacking histidine and supplemented with adenine (CSM-His w/Ade40), with 2% raffinose for a sugar source. Cells were diluted to an OD600 of 0.5 and then serial fivefold dilutions were prepared and added to plates containing CSM-His w/Ade40, 2% raffinose, 0.05% galactose to induce expression, and either 0mM, 10mM, or 20mM boric acid to challenge yeast growth. 10 μL of each dilution was plated, and plates were stored at 30°C for 4 days until imaged. The phylogenetic tree was generated using phylogeny.fr (Dereeper et al., 2008).

Western immunoblot analysis

Protein overexpression protocols were adapted from previously reported methods (Flores et al., 2019), but with the following distinctions: 50mL yeast cultures were grown using strain BTSY1 ( *MATα his3::GAL1-GAL4 pep4 prb1-1122* ), and after 16 h induction by 2% galactose the cells were lysed by passing once through an Emulsiflex-B15 homogenizer at 22,500 psi. Samples were spun for 10min at 10,000 x g, and then supernatants were collected and spun a second time for 1 h at 164,000 x g. The supernatants and pelleted membranes from this second, high-speed spin were used to run the gel for the Western immunoblot with 8.75μg total protein loaded per lane. Proteins were transferred to a PVDF membrane in a wet tank. The primary antibody was incubated at 4°C overnight, and the secondary antibody was incubated at room temperature for one hour before exposure using chemiluminescent horseradish peroxidase substrate. Total protein loading controls were imaged using Bio-Rad stain-free gels.

Borate quantification assay

The curcumin-based borate quantification assay was adapted from a previously published protocol (Mohan and Jones, 2018). Briefly, yeast colonies were used to inoculate overnight cultures which were then seeded to an OD600 of 0.25 the following morning and allowed to grow for 7 h until the OD600 was around 0.9. Protein expression was induced for 16 h by the addition of 2% galactose, after which cultures had 1mM boric acid added for 90min. Cells were pelleted and washed with water before being pelleted a second time, resuspended in 350μL water, and lysed by incubating at 98°C for 30min. After a 5min spin at 16,100 x g, 300μL of supernatant was prepared for curcumin addition per the previously reported protocol, with the data collected using a quartz cuvette that was washed twice with 91% isopropyl alcohol in between measurements. Standard curves were generated from standards containing 0, 0.625, 1.25, 2.5, and 5.0 mg/L of borate. All solutions in this experiment never touched glass to avoid the risk of borate entering the experiment via borosilicate glassware.

## Reagents

Table 1: Yeast strains used in this study

**Table:** 

Strain	Genotype	Figure	Source
BTSY1	*MATα his3::GAL1-GAL4 pep4 prb1-1122*	1C	Waight et al., 2013
BTSY2	*MATα his3::GAL1-GAL4 pep4 prb1-1122 bor1::KanMX*	1A, 1D	this study

Table 2: Antibodies used in this study

**Table:** 

Antibody description	Company, Cat#
anti-His _6_ , rabbit polyclonal	Abcam, ab9108
goat anti-rabbit secondary antibody, HRP-conjugated	Invitrogen, 32460

Table 3: Chemical reagents used in this study

**Table:** 

Name	Company, Cat#
Complete supplement mixture-His w/Ade40	Sunrise Science, 1023-100
Yeast nitrogen base + nitrogen	Sunrise Science, 1501-500
D(+)-Raffinose pentahydrate	Acros, 195675000
D(+)-Galactose	Acros, 15061-0010
Boric acid	Sigma, B6768
Difco™ agar	BD, 214530
4–20% Mini-PROTEAN® TGX Stain-Free™ Protein Gels	Bio-rad, 4568094
PVDF blotting sandwiches	Millipore, IPSN07852
SuperSignal™ West Pico PLUS chemiluminescent substrate	Thermo, 34577
Curcumin	Acros, 218580100
